# Quaternized Amphiphilic Block Copolymers/Graphene Oxide and a Poly(vinyl alcohol) Coating Layer on Graphene Oxide/Poly(vinylidene fluoride) Electrospun Nanofibers for Superhydrophilic and Antibacterial Properties

**DOI:** 10.1038/s41598-018-36479-w

**Published:** 2019-01-23

**Authors:** Jeong-Ann Park, Kie Yong Cho, Chee Hun Han, Aram Nam, Jae-Hyun Kim, Sang-Hyup Lee, Jae-Woo Choi

**Affiliations:** 1 Environmental Functional Materials and Water Treatment Laboratory, Department of Rural Systems Engineering, Seoul National University, Seoul, 08826 Republic of Korea; 2 Research Institute of Agriculture and Life Sciences, Seoul National University, Seoul, 08826 Republic of Korea; 3 Artie McFerrin Department of Chemical Engineering, Texas A&M University, College Station, TX 77843-3122 United States; 4 Center for Water Resource Cycle Research, Korea Institute of Science and Technology, Hwarangno 14-gil 5, Seongbuk-gu, Seoul 02792 Republic of Korea; 5 KU-KIST Green School, Graduate School of Energy and Environment, Korea University, 145 Anam-ro, Seongbuk-gu, Seoul 02841 Republic of Korea; 6 Division of Energy & Environment Technology, KIST School, Korea University of Science and Technology, Seoul, 02792 Republic of Korea

## Abstract

Poly(vinylidene fluoride) (PVDF) is common polymer for electrospinning, however, its high hydrophobicity is a major drawback, which cause fouling. To introduce hydrophilicity and antibacterial activity, quaternary ammonium-functionalized amphiphilic diblock copolymers were synthesized and blended with a PVDF/graphene oxide (GO) solution, then, electrospun and coated with a hydrophilic polymer, poly(vinyl alcohol) (PVA). The amphiphilic block copolymer, consisting of a hydrophobic poly(methyl methacrylate) block and a hydrophilic poly[N,N-2-(dimethylamino)-ethyl methacrylate) block (PMMA-*b*-PDMAEMA), was synthesized. Polymeric quaternary ammonium with three different alkyl chain lengths (C_2_, C_4_, and C_8_) were successfully introduced to obtain as *q*-PMMA-*b*-PDMAEMA. The *q*-PMMA-*b*-PDMAEMA in the nanofiber matrix was confirmed by C=O bands (1734 cm^−1^) in the Fourier transform infrared spectra. Nano-sized spherical protuberances were distributed on the surface as revealed by field emission scanning and transmission electron microscopies. The PVDF/GO/*q*-PMMA-*b*-PDMAEMA@PVA nanofibers has superhydrophilic properties (water contact angle = 0–20°) and the pure water flux was generally improved by increasing the alkyl chain length. When introducing the longest alkyl chain (C_8,OBC_), the total fouling ratio was the lowest (49.99%) and the bacteria removal capacities after 60 min were the highest for both *Escherichia coli* (4.2 × 10^5^ CFU/mg) and *Staphylococcus aureus* (6.1 × 10^5^ CFU/mg) via growth inhibition and cytoplasmic membrane damage.

## Introduction

Electrospun nanofibers have great potential to use in water treatment, such as large surface areas and high porosities, which are attributed to the 100–200 nm diameters of the fibers^1^. Poly(vinylidene fluoride) (PVDF) is one of the most common polymers for producing electrospun nanofibers used in microfiltration and ultrafiltration for industrial application owing to its high chemical resistance, good mechanical strength, thermal stability, and low cost^2,3^. However, the high hydrophobicity of PVDF is unsuitable for water treatment membranes, causing fouling, which results in reduced permeation fluxes and increased operating costs. To overcome this issue, many attempts have been made to improve the hydrophilicity of PVDF membranes, including hydrophilic polymer coating^4,5^, surface grafting^6^, blending amphiphilic polymers^7–10^, and introducing inorganic nanoparticles^11–14^. Surface grafting is one of simple and common chemical modification methods induced by plasma treatment, electron beam radiation, and O_3_/O_2_ pre-activation^3^, however, it could cause degradation of PVDF chains on the membrane surface due to introducing covalent bonding. On the other hand, coating a hydrophilic polymer layer on PVDF nanofibers is a simple and easy physical modification method to enhance hydrophilicity without changing chemical composition of PVDF; however, incompatibility has been observed between hydrophilic polymers and the hydrophobic PVDF matrix.

Amphiphilic polymers may be used as modifiers that have strong interactions with both the PVDF nanofiber matrix and hydrophilic polymers, such as poly(vinyl alcohol) (PVA)^9^. PVA is a promising hydrophilic polymer because of its high chemical resistance, thermal stability, low cost and non-toxicity to the environment^15,16^. Nevertheless, a hydrophilic surface alone is not sufficient to prevent fouling from biofilm formation, and antibacterial activity is also needed to overcome fouling.

Amphiphilic block copolymers, consisting of a hydrophobic block of poly(methyl methacrylate) (PMMA) and a hydrophilic block of poly[N,N-2-(dimethylamino) ethyl methacrylate) (PDMAEMA), have been investigated to enhance both the hydrophilic polymer compatibility and antibacterial activity of PVDF nanofiber^17–19^. MMA was chosen for the hydrophobic part owing to its high compatibility with PVDF for homogeneous blending and high retention^8^. In addition, quaternary ammonium compounds are well-known to have broad-spectrum of antimicrobial activity against bacteria, algae, and fungi via long alkyl chains^20^. Polymeric quaternary ammonium groups with long alkyl chains were introduced to the PMMA-*b*-PDMAEMA to yield *q*-PMMA-*b*-PDMAEMA amphiphilic block copolymers. It was synthesized by atom transfer radical polymerization (ATRP) for PMMA-*b*-PDMAEMA in combination with a Menshutkin reaction to introduce cationic quaternary ammonium groups^17–19^. Rationally designed amphiphilic copolymers with the aforementioned beneficial features could be desirable candidates in the engineering field for modification of PVDF membranes, enabling tunable hydrophilicity and favorable interfacial control^21–25^.

A few studies on the synthesis and blending of amphiphilic block copolymers with MMA and DMAEMA have been performed to improve the hydrophilicity and antibacterial activity of PVDF membranes using the phase-inversion method^8,9^. Sun *et al*.^9^ found that a PVDF/PMMA-*b*-PDMAEMA membrane slightly reduced protein adsorption and improved hydrophilicity compared with the pristine PVDF membrane. Kakihana *et al*.^8^ blended P(MMA-*co*-DMAEMA) containing quaternary ammonium groups in a PVDF membrane and showed a slight increase in antibacterial activity against *Escherichia coli* with one alkyl chain, however, the modified membrane exhibited a more hydrophobic nature than the pristine PVDF membrane. Therefore, two previous studies provided limited results of improving both hydrophilicity and antibacterial activity of PVDF membrane. To the best of our knowledge, blends of PVDF nanofibers with amine quaternized PMMA-*b*-PDMAEMA diblock copolymers (*q*-PMMA-*b*-PDMAEMA) with alkyl chains have not been investigated for enhancing anti-fouling activity through increased hydrophilicity owing to PVA coating layers and increased antibacterial activity by quaternary ammonium compounds (QACs). In our previous study^26^, graphene oxide (GO)/PVDF nanofibers showed better tensile strength, thermal stability, and antibacterial property than those of PVDF nanofibers, however, hydrophilicity is slightly enhanced. Generally, GO into PVDF membrane has been mainly studied to increase pollutant adsorption capacity^27,28^, improve antibacterial, and mechanical strength^29^, moreover, it can prevent the release of QACs from PVDF membranes, and achieve better QAC stability in membranes without sacrificing antibacterial activity^30^.

Therefore, the aim of this study was to synthesize PVDF/GO nanofibers containing *q*-PMMA-*b*-PDMAEMA using electrospinning technology (PVDF/GO/*q*-PMMA-*b*-PDMAEMA nanofibers) to achieve high anti-fouling activity induced by superhydrophilicity and anti-bacterial properties. Specifically, 1) the synthesis of *q*-PMMA-*b*-PDMAEMA with different alkyl chain lengths using CRP-ATRP; 2) the preparation and characterization of PVDF/GO/*q*-PMMA-*b*-PDMAEMA nanofibers with PVA coating layer for microfiltration; and 3) the evaluation of anti-fouling activity using fouling reversibility test and anti-bacterial experiments (agar diffusion, dynamic contact, and bacterial cell viability) were investigated.

## Results and Discussion

### Synthesis and characterization of quaternary ammonium-functionalized amphiphilic diblock copolymers (q-PMMA-b-PDMAEMA)

**Figure 1:**
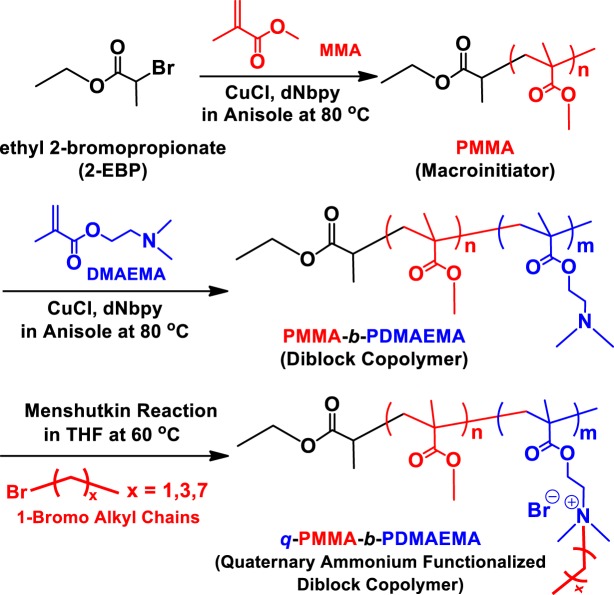
Synthesis of quaternary ammonium-functionalized amphiphilic block copolymers (*q-*PMMA-*b*-PDMAEMA).

Quaternary ammonium-functionalized amphiphilic diblock copolymers were synthesized by a combination of Cu-catalyzed ATRP for PMMA-*b*-PDMAEMA diblock copolymers and the Menshutkin reaction for functionalization of the PDMAEMA block using 1-bromo alkyl chains of varying length of alkyl chains (from ethane to octane) (Fig. 1). The acquired PMMA macroinitiator was examined by GPC to determine the molecular weight and molecular weight distribution, exhibiting 9,500 g/mol and 1.17, respectively (Fig. S1). After the copolymerization of DMAEMA to produce PMMA-*b*-PDMAEMA diblock copolymers, the GPC trace smoothly shifted to lower elution volumes, indicating an increase in molecular weight (Fig. S1). In addition, the GPC curve of PMMA-*b*-PDMAEMA was as narrow as that of PMMA, corresponding to a PDI of 1.21 (Fig. S1).

**Figure 2:**
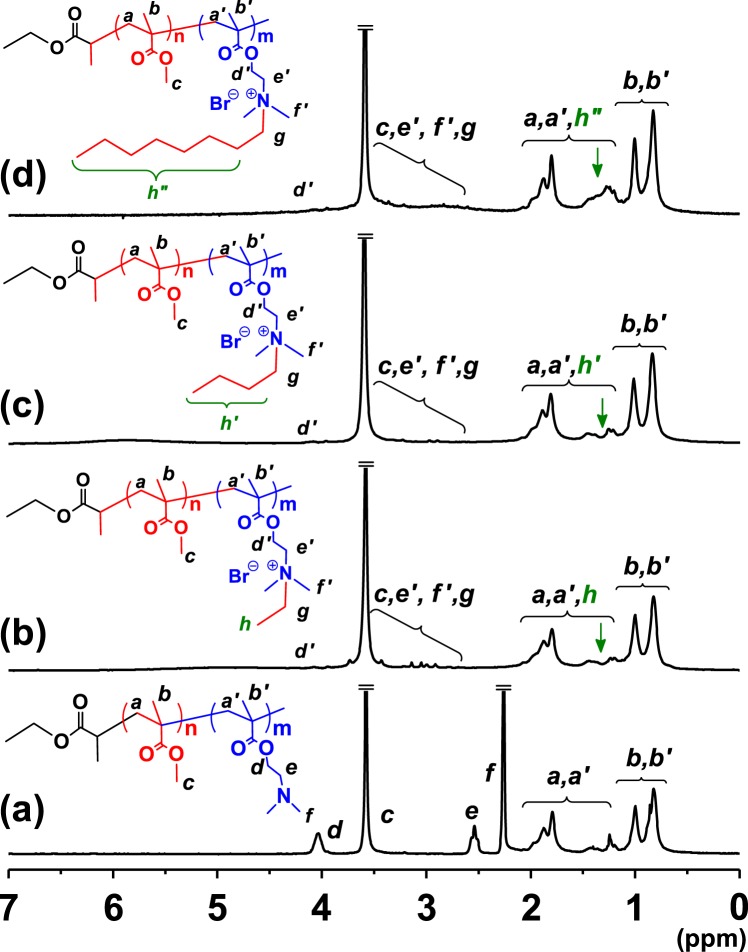
^1^H NMR spectra of (**a**) PMMA-*b*-PDMAEMA, (**b**) ethyl-PMMA-*b*-PDMAEMA (EBC), (**c**) butyl-PMMA-*b*-PDMAEMA (BBC), and (**d**) octyl-PMMA-*b*-PDMAEMA (OBC) amphiphilic block copolymers in CDCl_3_ at 25 °C.

The block fraction and chemical structures of the acquired amphiphilic diblock copolymers were characterized by ^1^H NMR spectroscopy (Fig. 2). The ^1^H NMR characteristics provided the weight fraction of PMMA and PDMAEMA which was calculated to be 0.66 and 0.34, respectively, using the integration of *d* and *c* peaks (Fig. 2a). The ^1^H NMR spectrum of PMMA-*b*-PDMAEMA exhibited typical ester methylene peaks at 3.56 (*c*) and 4.03 (*d*) ppm, which corresponding to PMMA and PDMAEMA, respectively (Fig. 2a). In addition, the dimethylamino groups in the PDMAEMA segment led to sharp peaks at 2.54 and 2.26 ppm derived from the two protons of methylene (*e*) and six protons of dimethyl (*f*) groups, respectively (Fig. 2a). The presence of 1-bromo alkyl chains to the PDMAEMA segment afforded substantial downfield shifts and broadening of the *e* and *f* peaks owing to the formation of quaternary ammonium groups. These peak changes following quaternization of the PDMAEMA segment are consistent with previous reports on pyrene functionalization of PDMAEMA by the Menshutkin reaction^17^. Thus, the almost complete disappearance of peaks at *e* and *f* indicated that the reaction was performed high yield (>99% conversion) reaction was performed. Furthermore, the presence of alkyl chains onto the dimethylamino groups resulted in new broad peaks in the range of 1.13–1.54 ppm, which are denoted as *h*, *h’*, and *h”* (Fig. 2b–d). The introduction of longer alkyl chains ligated at the PDMAEMA segment resulted in more intense and broader peaks, as indicated by a green arrow in Fig. 2b–d. Thus, quaternary ammonium groups were successfully synthesized in *q*-PMMA-*b*-PDMAEMA.

### Characterization of PVDF/GO/q-PMMA-b-PDMAEMA@PVA nanofibers

**Figure 3:**
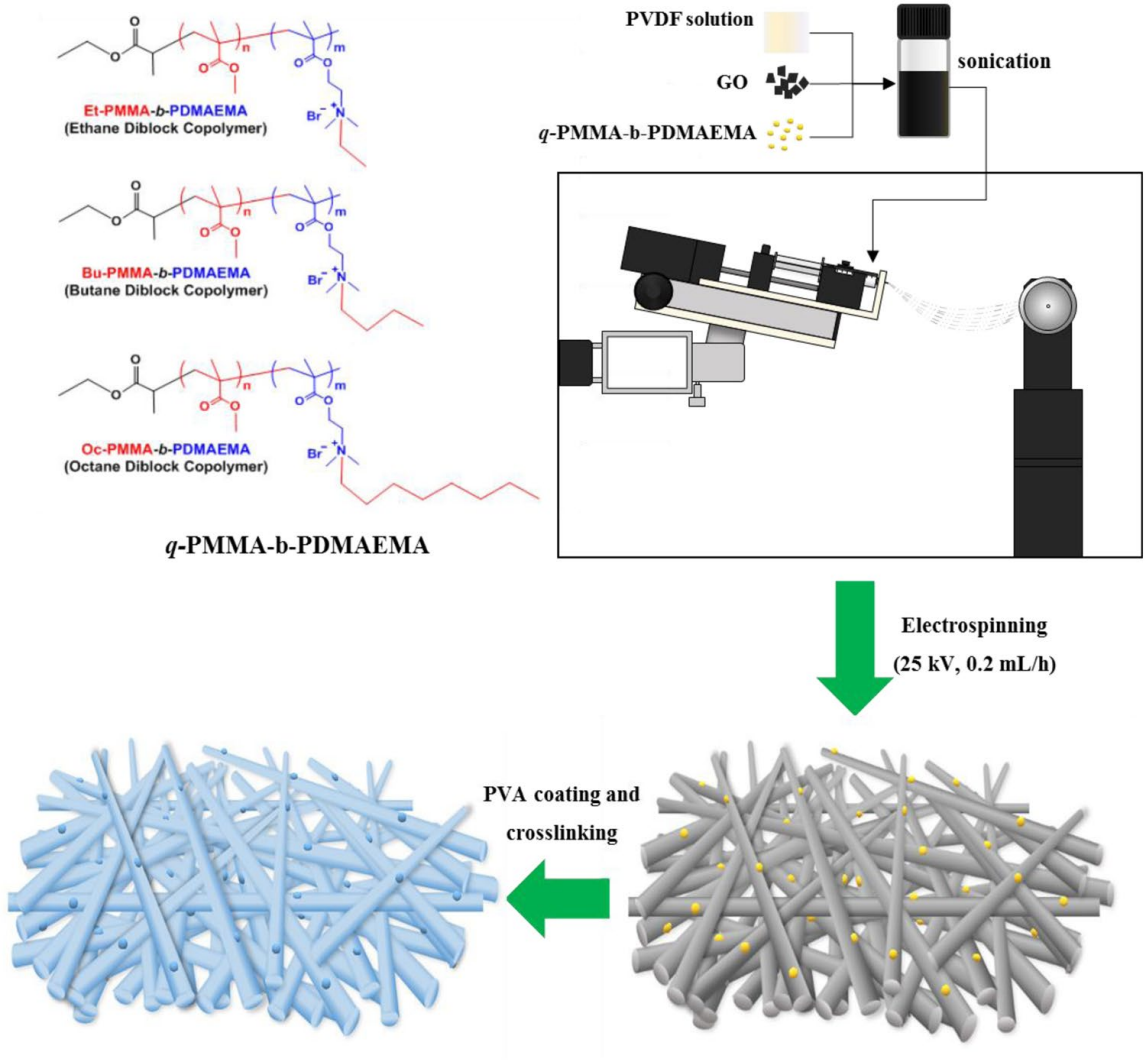
Schematic illustration of the synthesis of PVDF/GO/*q*-PMMA-*b*-PDMAEMA@PVA nanofibers.

**Figure 4:**
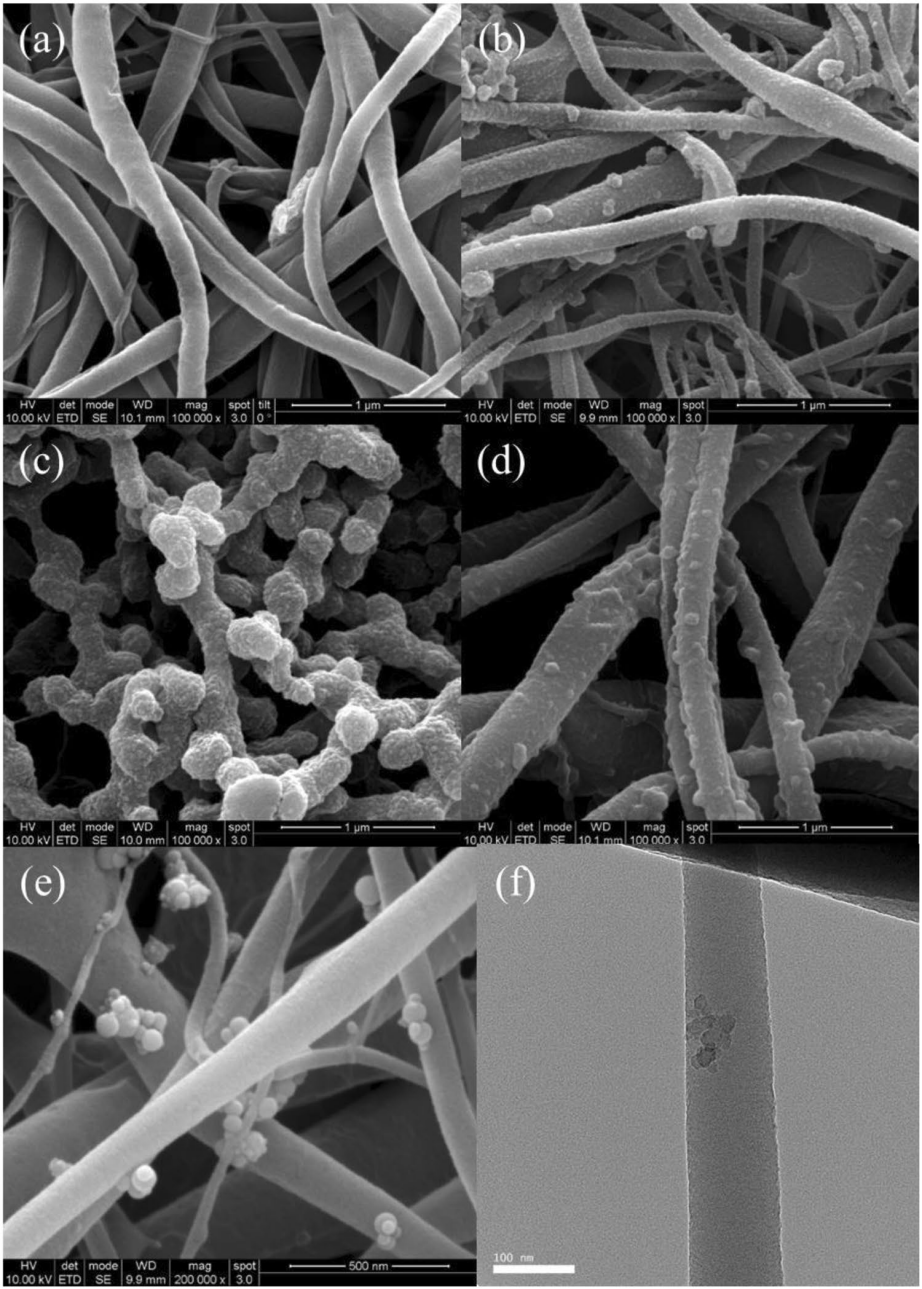
FESEM images of (**a**) PVDF/GO@PVA, (**b**) PVDF*/*GO/EBC@PVA, (**c**) PVDF*/*GO/BBC@PVA, (**d**) PVDF/GO/OBC@PVA, and (**e**) PVDF/GO/OBC nanofibers, and (**f**) TEM image of PVDF/GO/OBC nanofibers.

Figure 3 illustrates the entire modification process for producing PVDF/GO/*q*-PMMA-*b*-PDMAEMA@PVA nanofibers. The obtained nanofibers were characterized in detail. FESEM images (Fig. 4) show the morphologies of PVDF/GO@PVA (Fig. 4a), PVDF/GO/EBC@PVA (Fig. 4b), PVDF/GO/BBC@PVA (Fig. 4c), and PVDF/GO/OBC@PVA (Fig. 4d) nanofibers. With the addition of *q*-PMMA-*b*-PDMAEMA, nano-sized spherical protuberances are observed, which are well distributed on the surface of each nanofiber. The PVDF/GO/OBC nanofibers exhibited clear spherical OBC structures, as observed by FESEM (Fig. 4e) and TEM (Fig. 4f). Subsequent coating with a crosslinked PVA layer on the surface prevented the release of OBC from the PVDF/GO/OBC nanofibers (Fig. 4d).

**Figure 5:**
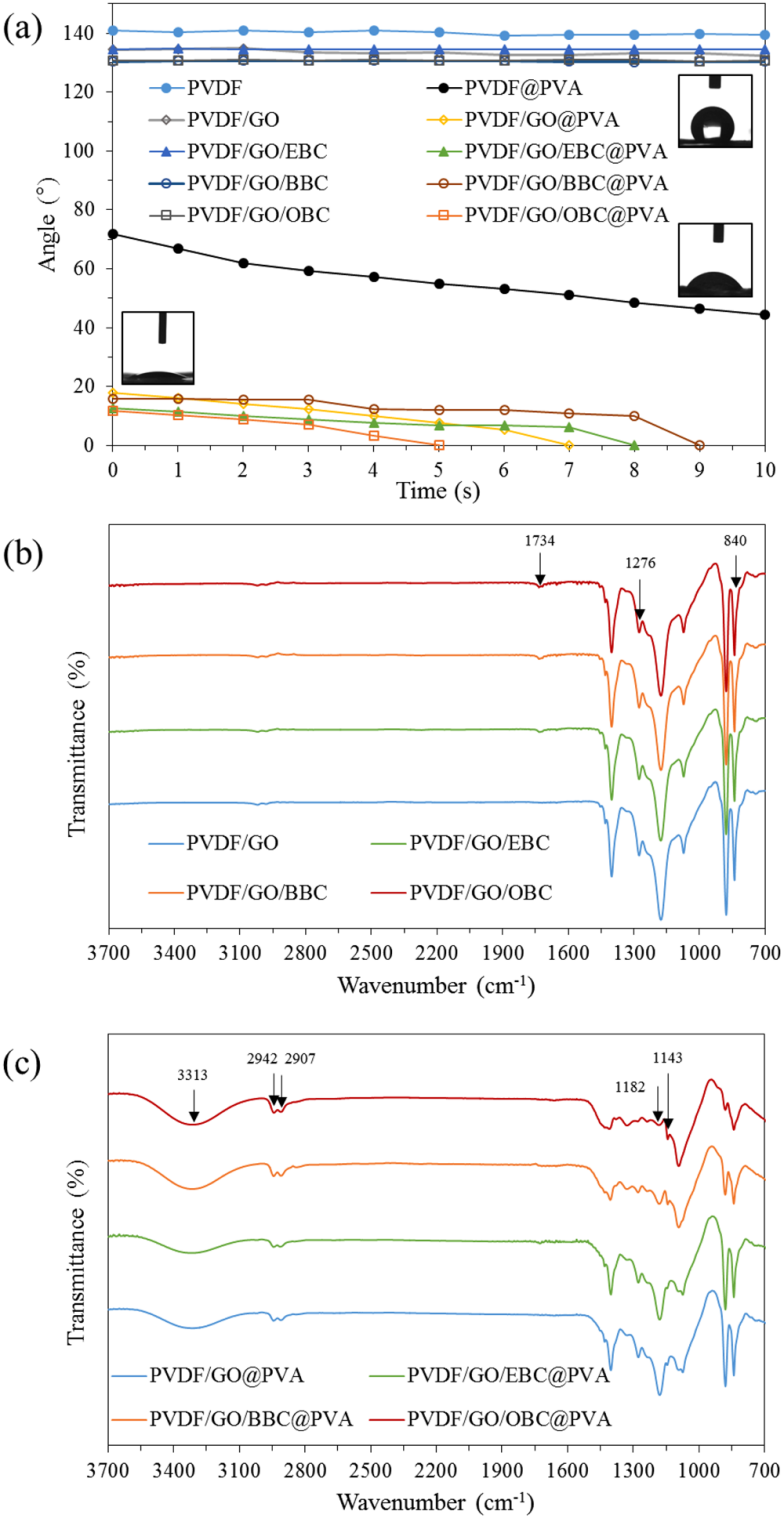
(**a**) Water contact angles of PVDF, PVDF/GO, PVDF/GO/EBC, PVDF/GO/BBC, PVDF/GO/OBC, PVDF/GO@PVA, PVDF/GO/EBC@PVA, PVDF/GO/BBC@PVA and PVDF/GO/OBC@PVA nanofibers at 10 s and (**b**) FT-IR spectra of PVDF/GO, PVDF/GO/EBC, PVDF/GO/BBC, PVDF/GO/OBC and (**c**) FT-IR spectra of PVDF/GO@PVA, PVDF/GO/EBC@PVA, PVDF/GO/BBC@PVA and PVDF/GO/OBC@PVA nanofibers.

The average diameters of each of the nanofibers was in the range of 141.2–181.6 nm, as summarized in Table 1. The pure water permeation flux was enhanced by the addition of *q*-PMMA-*b*-PDMAEMA, with the highest pure water flux (2495.0 LMH/bar) obtained for PVDF/GO/OBC@PVA nanofibers, which had the lowest water contact angle of 11.6° (Fig. 5a). Before PVA coating, the water contact angles of PVDF, PVDF/GO, PVDF/GO/EBC, PVDF/GO/BBC, and PVDF/GO/OBC were all very high (140.9°, 134.9°, 134.4°, 130.9°, and 130.6°, respectively, at 2 s), which indicates that these nanofibers were highly hydrophobic, even with the amphiphilic copolymer *q*-PMMA-*b*-PDMAEMA. Kakihana *et al*.^8^ examined a quaternary-ammonium containing P(MMA-co-DMAEMA)/PVDF membrane, which still showed a high water contact angle of 132° (PVDF membrane = 110°). Sun *et al*.^9^ showed that water contact of PVDF/PMMA-*b*-PDMAEMA membrane was still high as 76° compared with that of pristine PVDF membrane (98°). However, the water contact angles of the PVDF/GO@PVA, PVDF/GO/EBC@PVA, PVDF/GO/BBC@PVA, and PVDF/GO/OBC@PVA nanofibers were less than 20° at 1 s and finally 0° within 9 s via PVA coating and crosslinking step, indicating that the nanofibers became superhydrophilic. By methanol treatment, the PVA based nanofibers were stable in aqueous solution because residual water within the nanofibers were removed, which enhancing intermolecular polymer hydrogen bonding instead of PVA-water hydrogen bonding^31^. Similarly, Lu *et al*.^5^ found that a PVDF membrane coated with a PVA layer had a superhydrophilic nature. The incorporation of GO and *q*-PMMA-*b*-PDMAEMA can enhance PVA attachment on PVDF nanofibers compared with that on pristine PVDF nanofibers, which still had a water contact angle of 62° even coating with crosslinked PVA. The amounts of PVA coated on PVDF/GO, PVDF/GO/EBC, PVDF/GO/BBC, and PVDF/GO/OBC nanofibers were 0.043 ± 0.000 g/g, 0.063 ± 0.000 g/g, 0.062 ± 0.001 g/g, and 0.109 ± 0.000 g/g, respectively. Therefore, *q*-PMMA-*b*-PDMAEMA with longer alkyl chains tended to lower the water contact angle by increasing the attachment of the PVA layer.

**Table 1:** Characteristics of PVDF/GO@PVA and PVDF/GO/*q*-PMMA-*b*-PDMAEMA@PVA nanofibers.

Type of nanofibers	Pure water flux (LMH/bar)	Average diameter of nanofibers (nm)	Zeta potential at pH 6 (mV)	Initial water contact angle (°) (0 s)	Porosity (%)	Mean flow pore diameter (μm)	Smallest flow pore diameter (μm)	Largest flow pore diameter (μm)
PVDF/GO@PVA	979.9	176.0 ± 14.9	0.31 ± 0.11	17.9	80.74	0.36	0.17	0.96
PVDF*/*GO/EBC@PVA	1175.9	141.2 ± 82.4	−1.56 ± 0.54	12.7	71.66	0.41	0.33	0.47
PVDF*/*GO/BBC@PVA	1763.8	181.6 ± 95.6	−1.26 ± 0.33	15.9	76.25	0.23	0.08	0.54
PVDF/GO/OBC@PVA	2495.0	164.2 ± 97.8	−1.04 ± 0.10	11.6	71.67	0.45	0.18	0.73

As the zeta potential values of PVDF/GO and PVDF/GO@PVA nanofibers are 1.26 and 0.31 mV, respectively, those of PVDF/GO/*q*-PMMA-*b*-PDMAEMA@PVA nanofibers can be predicted to be more positive owing to the effect of cationic charged groups (N^+^) in *q*-PMMA-*b*-PDMAEMA. However, the zeta potentials of the PVDF/GO/*q*-PMMA-*b*-PDMAEMA@PVA nanofibers were negative (−1.04 to −1.56 mV) because of the relatively high negative charge of the PVA (−4.7 mV) coating layer, which was bound more by *q*-PMMA-*b*-PDMAEMA, especially OBC.

FT-IR spectra were collected before (Fig. 5b) and after PVA coating (Fig. 5c). PVDF is a semicrystalline polymer and has five crystalline phases including α, β, γ, δ, and ε, of which α and β are the most common and the *β*-phase are the highest dipolar contributor among other phases^32^. The *β*-phase was dominantly observed with the strong peaks at 840 and 1276 cm^−1^ of all PVDF/GO/*q*-PMMA-*b*-PDMAEMA nanofibers (Fig. 5b)_._ The calculated F(β) values of pristine PVDF, PVDF/GO, PVDF/GO/EBC, PVDF/GO/BBC and PVDF/GO/OBC nanofibers were 83.6, 87.7, 87.0, 87.5, and 87.2%, respectively. This indicating that *β*-phase is predominant of all PVDF/GO/*q*-PMMA-*b*-PDMAEMA nanofibers attributed by electrospinning process (electrical field and stretching forces) and addition of GO (1.5 wt%); however, *q*-PMMA-*b*-PDMAEMA (0.5 wt%) showed a little impact on phase conformation of PVDF. Similarly, Sharma *et al*.^33^ insisted that PVDF electrospun nanofibers predominantly consisted of *β*-phase of PVDF because high-voltage conditions caused extensional forces on the PVDF chains to aligns the dipoles in one direction; also, incorporation of carbon nanotubes induce *β*-phase of PVDF due to charge accumulation. Abbasipour *et al*.^34^ reported that α-phase decreased dramatically by transforming the PVDF powder to the nanofiber and addition of GO in PVDF nanofibers enhanced the *β*-phase of PVDF via enhancing dipole-dipole forces. On the other hand, Cui *et al*.^35^ suggested that PMMA grafted TiO_2_ inhibited the orientation of PVDF chains during the co-electrospinning process leading decrease of *β*-phase content. A peak at 1734 cm^−1^ was only observed for in PVDF/GO/EBC, PVDF/GO/BBC and PVDF/GO/OBC nanofibers, not for PVDF/GO nanofibers (Fig. 5b). Thus, this peak was attributed to the C=O bonds in *q*-PMMA-*b*-PDMAEMA. Three peaks at 3313, 2942 and 2907 cm^−1^ were clearly observed for all the nanofibers (Fig. 5c), corresponding to O-H vibration and C-H stretching in the PVA coating layer^36^. The FT-IR spectrum of PVDF/GO/OBC@PVA nanofibers showed clear additional peaks at 1182 and 1143 cm^−1^, which were attributed to C-O stretching and C-C-C stretching in the PVA coating layer. Therefore, as indicated by various characterization techniques, the PVA layer is more favorably coated on the PVDF/GO/OBC nanofibers than on the other nanofiber.

The largest flow pore sizes for the PVDF/GO@PVA, PVDF/GO/EBC@PVA, PVDF/GO/BBC@PVA, and PVDF/GO/OBC@PVA nanofibers were less than 1 μm, and the mean flow pore diameters were in the range of 0.23–0.45 μm (Table 1). The pure water flux generally improved with increasing alkyl chain length owing to increase hydrophilicity, as indicated by a decrease in the water contact angle (Fig. 5a). The porosity of the PVDF/GO@PVA nanofibers was 80.74%, which is slightly higher than those of PVDF/GO/EBC@PVA (71.66%), PVDF/GO/BBC@PVA (76.25%), and PVDF/GO/OBC@PVA (71.67%) nanofibers; however, the porosities of all these nanofibers are still sufficiently high for microfiltration.

### Anti-fouling activity

**Figure 6:**
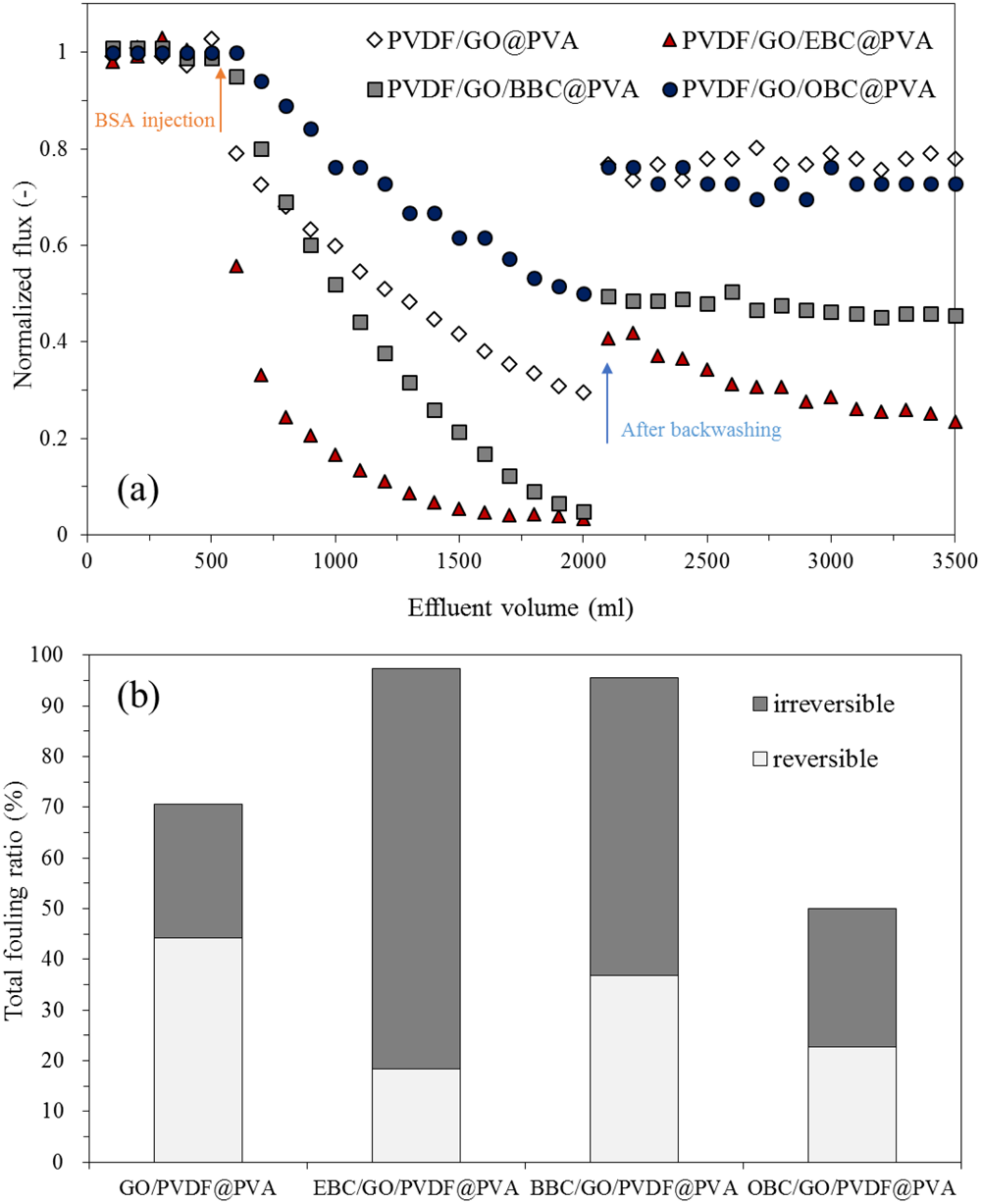
Anti-fouling activity of PVDF/GO/*q*-PMMA-*b*-PDMAEMA@PVA nanofibers: (**a**) normalized flux before and after BSA filtration; and (**b**) total fouling ratio (%) including reversible and irreversible fouling.

The anti-fouling performance of the PVDF/GO/*q*-PMMA-*b*-PDMAEMA@PVA nanofibers was investigated by measuring the normalized water flux before and after injection of a BSA solution as a model protein foulant (Fig. 6). After injection of 1.5 L of BSA solution (1 g/L), the normalized fluxes of PVDF/GO/EBC@PVA, PVDF/GO/BBC@PVA and PVDF/GO@PVA nanofibers decreased steeply to 0.03, 0.05, and 0.29, respectively, whereas that of the PVDF/GO/OBC@PVA nanofibers was only reduced to 0.52 (Fig. 6a). The total fouling ratio was high, decreasing in the order of PVDF/GO/EBC@PVA (97.26%) > PVDF/GO/BBC@PVA (95.60%) > PVDF/GO@PVA (70.54) > PVDF/GO/OBC@PVA (49.99%) nanofibers (Fig. 6b).

In addition, 45.5% of the total fouling was reversible fouling for the PVDF/GO/OBC@PVA nanofibers. The highest BSA adsorption amount was calculated as 13.18 mg/cm^2^ for the PVDF/GO@PVA nanofibers, whereas the BSA adsorption amount was drastically decreased by the incorporation of *q*-PMMA-*b*-PDMAEMA (OBC: 4.61 mg/cm^2^, BBC: 4.37 mg/cm^2^, and EBC: 1.66 mg/cm^2^), which corresponded to the trend observed for the zeta potentials. A negatively charged nanofiber surface can effectively inhibit protein adsorption. The anti-fouling properties were mainly enhanced by increasing the alkyl chain length among PVDF/GO/*q*-PMMA-*b*-PDMAEMA@PVA nanofibers with minor effects attributed to surface charge and flow pore diameters. All the nanofibers had similar mean flow pore diameters (0.23–0.45 μm), however, the PVDF/GO@PVA nanofibers had the largest pore diameter (0.96 μm); thus, BSA blockage was decreased relative to the other nanofibers and this could be easily backwashed. After backwashing with deionized water, the normalized flux of the PVDF/GO/OBC@PVA nanofibers was recovered to over 0.7, whereas those of the PVDF/GO/EBC@PVA and PVDF/GO/BBC@PVA nanofibers were 0.45 and 0.25, respectively. For PVDF/GO/OBC@PVA nanofibers, the increased hydrophilicity and slightly negatively charged surface owing to the long alkyl chain of OBC and the PVA layer successfully prevent BSA adsorption and enhance BSA desorption.

### Anti-bacterial activity

The results of the agar diffusion method using PVDF/GO/*q*-PMMA-*b*-PDMAEMA@PVA nanofibers are summarized in Table S1. A slight zone of inhibition was observed around PVDF/GO/BBC@PVA nanofibers using *E*. *coli* and PVDF/GO/OBC@PVA nanofibers using *E*. *coli* and *S*. *aureus*. All bacteria grew well under the PVDF/GO@PVA nanofibers, whereas no growth of *E*. *coli* and *S*. *aureus* was observed when in direct contact with the PVDF/GO/OBC@PVA nanofibers. Antibacterial effects are enhanced by increasing the length of alkyl moieties, which strongly react with the cytoplasmic membranes of cells^37,38^. This finding demonstrated that *q*-PMMA-*b*-PDMAEMA could not diffuse to and be released into the agar owing to strong incorporation into the nanofibers, which maintained durable antibacterial activity. Thus, PVDF/GO/OBC@PVA nanofibers effectively inhibit the growth of bacteria.

**Figure 7:**
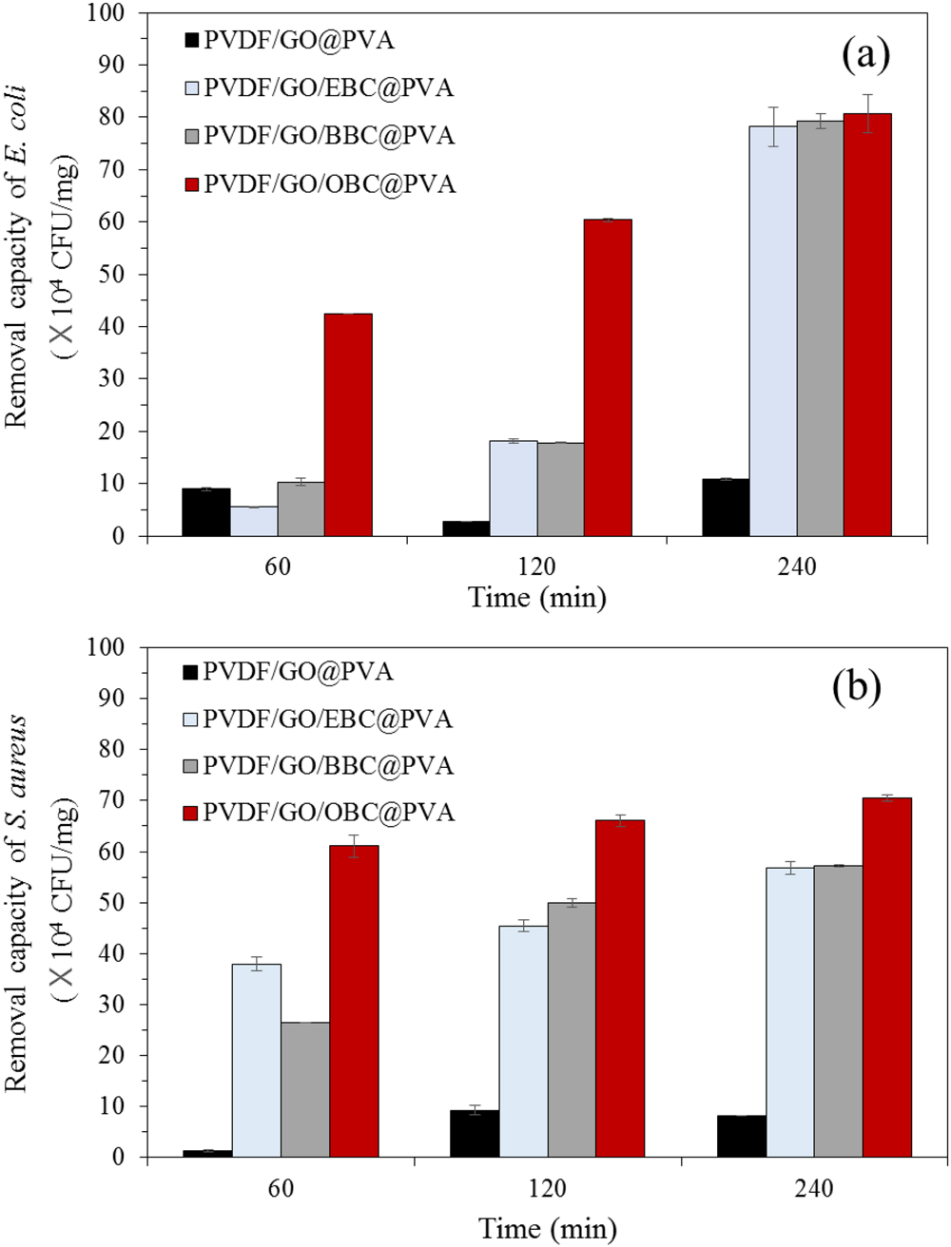
Bacteria removal capacity (CFU/mg) of PVDF/GO@PVA, PVDF/GO/EBC@PVA, PVDF/GO/BBC@PVA and PVDF/GO/OBC@PVA nanofibers: (a) *E*. *coli* and (b) *S*. *aureus*. Error bars represent the standard deviation of the mean (n = 4).

The results of the dynamic contact tests, as shown in Fig. 7a (*E. coli*) and 7b (*S*. *aureus*), indicating that the incorporation of *q*-PMMA-*b*-PDMAEMA greatly enhanced the *E*. *coli* and *S*. *aureus* removal capacities compared with the minimum effect of PVDF/GO@PVA nanofibers. The removal capacity is assumed to be enhanced by two mechanisms: 1) positively charged ammonium ions on *q*-PMMA-*b*-PDMAEMA could attract negatively charged bacteria to the surface of the nanofibers and 2) cell damage by alkyl chains on *q*-PMMA-*b*-PDMAEMA could occur. The removal capacities increased as the length of the alkyl moieties increased, similar to the results of the agar diffusion method. Of the examined samples, the PVDF/GO/OBC@PVA nanofibers were the most effective for the removal of both *E*. *coli* (4.2 × 10^5^ CFU/mg) and *S*. *aureus* (6.1 × 10^5^ CFU/mg) after 60 min, and the removal capacities increased with increasing contact time. Similarly, Lin *et al*.^39^ found that the antibacterial properties against *E*. *coli* and *S*. *aureus* were increased by increasing the length of alkyl chains coated on cotton fabric from C_12_ to C_18_. Rawlinson *et al*.^40^ investigated the minimum inhibition concentration (MIC) of PDMAEMA using 13 bacteria species and found that it had antimicrobial effects on both gram-negative (MIC = 0.1–1 mg/mL) and gram-positive bacteria (MIC = 0.1–18 mg/mL).

**Figure 8:**
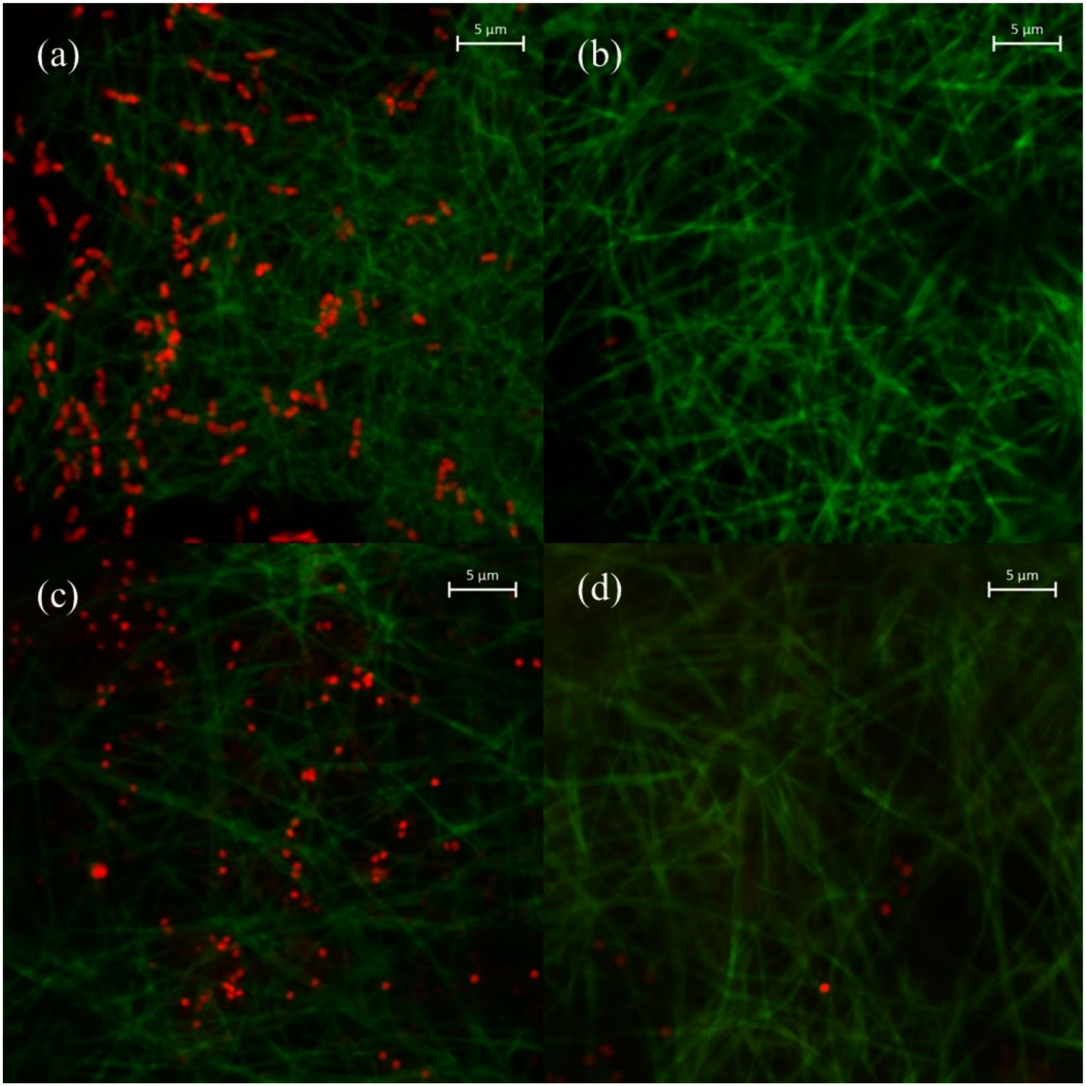
Fluorescence microscopy images (scale bar = 5 μm) of bacteria on nanofibers: (**a**) *E*. *coli* on PVDF/GO/OBC@PVA nanofibers, (**b**) *E*. *coli* on PVDF/GO@PVA nanofibers, (**c**) *S*. *aureus* on PVDF/GO/OBC@PVA nanofibers, and (**d**) *S*. *aureus* on PVDF/GO@PVA nanofibers.

LIVE/DEAD bacterial viability assays were conducted and observed using CLSM to verify the mechanism of bacteria removal by PVDF/GO/OBC@PVA nanofibers compared with that of PVDF/GO@PVA nanofibers (Fig. 8). In these images, the nanofibers are shown as green and a number of dead bacterial cells (*E*. *coli* and *S*. *aureus*), which were stained red, were clearly observed on the PVDF/GO/OBC@PVA nanofibers (Fig. 8a,c), however, a few dead bacterial cells were observed on the PVDF/GO@PVA nanofibers (Fig. 8b,d). This result indicated that *E*. *coli* and *S*. *aureus* were removed by bacterial cell death via cytoplasmic membrane damage attributed to OBC. Kakihana *et al*.^8^ suggested that *E*. *coli* was killed by a quaternized P(MMA-*co*-DMAEMA)/PVDF membrane because DMAEMA could penetrate the outer membrane and disrupt the cytoplasmic membrane. Park *et al*.^41^ showed CLSM images to verify that benzyl triethylammonium chloride (a type of QAC)/PVA nanofibers could damage the cytoplasmic membranes of *K*. *pneumoniae*, *E*. *coli* and *S*. *aureus*. Chen *et al*.^42^ visualized the bacteria on a dodecyl dimethyl benzyl ammonium chloride (a type of QAC)/PVDF membrane using CLSM, showing that the membrane killed bacteria already attached to the surface, which led to a reduction of biofilm. Thus, PVDF/GO/OBC@PVA nanofibers can improve the anti-fouling activity, as contact with these nanofibers can inhibit bacterial growth and cause cytoplasmic cell membrane damage.

Two modifications of PVDF nanofibers, blending with quaternary ammonium-functionalized amphiphilic block copolymers and PVA coating, were explored to overcome fouling via introducing superhydrophilic surfaces and antibacterial activity. These modifications effectively increased the hydrophilicity and pure water flux, while maintaining high porosity. The synthesized PVDF/GO/OBC@PVA nanofibers displayed a low total fouling ratio and inhibited protein adsorption, and the initial flux was recovered well after backwashing. In addition, the PVDF/GO/OBC@PVA nanofibers showed antibacterial activity against both gram-positive and gram-negative bacteria, as contact inhibited bacterial growth and caused cytoplasmic cell membrane damage. Therefore, the results highlight the potential use of the developed anti-fouling nanofibers for application in water purification.

## Conclusions

In this work, two modifications of PVDF nanofibers, blending with quaternary ammonium-functionalized amphiphilic block copolymers and PVA coating, were explored to overcome fouling via introducing superhydrophilic surfaces and antibacterial activity. These modifications effectively increased the hydrophilicity and pure water flux, while maintaining high porosity. The synthesized PVDF/GO/OBC@PVA nanofibers displayed a low total fouling ratio and inhibited protein adsorption, and the initial flux was recovered well after backwashing. In addition, the PVDF/GO/OBC@PVA nanofibers showed antibacterial activity against both gram-positive and gram-negative bacteria, as contact inhibited bacterial growth and caused cytoplasmic cell membrane damage. Therefore, the results highlight the potential use of the developed anti-fouling nanofibers for application in water purification.

## Materials and Methods

### Synthesis of PMMA-b-PDMAEMA amphiphilic diblock copolymers

All reagents were purchased from Alfa Aesar and Sigma Aldrich. According to the typical ATRP technique, all reagents, CuCl (0.78 mM, 77.7 mg), anisole (10.5 mL), 4,4′-di(5-nonyl)-2,2′-bipyridyl (dNbpy) (1.6 mM, 0.64 mg), MMA (56 mM, 6.0 mL), and a solution of ethyl 2-bromopropionate (0.6 mM, 0.9 mL, 700 mM in toluene), were sequentially added to a baked reaction flask under Ar. The polymerization was allowed to proceed by heating at 80 °C for 14 h. The reaction conversion (97%) was determined from the ^1^H NMR spectrum of the reaction solution using signals corresponding to the polymer and the unreacted monomer. The reaction was terminated by cooling to −78 °C using liquid nitrogen. The quenched solution was then diluted with tetrahydrofuran (THF) (10 mL) and then passed through an aluminum oxide column. The solution was collected, and solvents were removed under reduced pressure. The concentrated solution was dropped into methanol. The obtained white precipitator was collected by filtration and then dried at 25 °C for 48 h under vacuum. Sequential copolymerization with PDMAEMA block was carried out using the PMMA as a macroinitiator. The synthesis procedure for the PDMAEMA second block was the same as the process used to synthesizes PMMA. All reagents, CuCl (0.63 mM, 62.1 mg), anisole (8.3 mL), dNbpy (1.3 mM, 0.51 mg), PMMA (0.5 mM, 1 g), and DMAEMA (15 mM, 2.54 mL), were sequentially added to a baked 50 mL flask under Ar. The reaction was performed by stirring at 80 °C for 12 h. The resultants polymers exhibited 93% conversion. The reaction solution was quenched by immersing in liquid nitrogen. The purification procedure was the same as the method used for PMMA. The solvent for precipitation was only changed to hexane. ^1^H NMR (300 MHz, CDCl_3,_ δ) (ppm): 4.03 (s, 2 H, -OCH_2_-), 3.56 (s, 3 H, -OCH_3_), 2.54 (t, 2 H, -CH_2_N-), 2.26 (s, 6 H, -N(CH_3_)_2_), 1.2–2.1 (broad, 2 H, -CH_2_C- backbone), 0.7–1.1 (broad, 3 H, -CCH_3_ backbone).

### Synthesis of quaternary ammonium-functionalized amphiphilic diblock copolymers (q-PMMA-b-PDMAEMA)

Based on previously reported protocols, the obtained PMMA-*b*-PDMAEMA diblock copolymers were functionalized using the Menshutkin reaction to introduce with 1-bromo linear alkyl chains into the PDMAEMA block^17,18^. For this, PDMAEMA-*b*-PMMA (5 mM, 0.5 g) was dissolved in THF (7.7 mL). Thereafter, 1-bromoethane (0.17 M, 0.1 mL), 1-bromobutane (0.17 M, 0.14 mL), or 1-bromooctane (0.17 M, 0.226 mL) was added each to the above solution. The reaction was allowed to proceed by stirring at 60 °C for 24 h, yielding ca. 99% conversion and over 30 alkyl groups per chain. Purification was performed by precipitation in 300 mL of hexane after dilution with THF (10 mL). The three acquired *q*-PMMA-*b*-PDMAEMA resultants, ethyl-PMMA-*b*-PDMAEMA (EBC), butyl-PMMA-*b*-PDMAEMA (BBC), and octyl-PMMA-*b*-PDMAEMA (OBC), were dried at 25 °C for 48 h under reduced pressure. ^1^H NMR (300 MHz, CDCl_3,_ δ) (mg/L): 3.50–4.30 (broad, 2 H, -OCH_2_-), 3.56 (s, 3 H, -OCH_3_), 2.50–3.60 (broad, 2 H, -CH_2_N-), 2.50–3.60 (broad, 6 H, -N(CH_3_)_2_), 1.2–2.1 (broad, 2 H, -CH_2_C- backbone), 1.13–1.54 (broad, functionalized alkyl chains, -NCH_2_(CH_2_)_x_, x = 1, 3, 7), 0.7–1.1 (broad, 3 H, -CCH_3_ backbone). The number-average molecular weight (*M*_n_) and molecular weight distribution (*M*_w_/*M*_n_) of each samples were measured using a JASCO PU-2080 plus SEC system equipped with RI-2031 and UV-2075 (254 nm detection wavelength) detectors using THF as the eluent at 40 °C with a flow rate of 1 mL/min. The samples were separated using four columns: Shodex-GPC KF-802, KF-803, KF- 804, and KF-805. ^1^H NMR spectra were recorded in CDCl_3_ at 25 °C on a 300 MHz Varian Unity INOVA spectrometer.

### Preparation of PVDF/GO/q-PMMA-b-PDMAEMA@PVA nanofibers

Electrospun PVDF/GO/*q*-PMMA-*b*-PDMAEMA nanofibers were fabricated by an electrospinning system (ESR200R2, NanoNC, Seoul, Korea) at room temperature and relative humidity of 25–30%. Based on prior experiments to examine the effect of various applied voltage (10–30 kV) and flow rate (0.1–1.5 ml/h), the optimum electrospinning conditions of PVDF/GO/*q*-PMMA-*b*-PDMAEMA solutions were determined as 0.2 mL/h of flow rate, 25 kV of applied voltage, and 15 cm of tip-to-collector distance. The PVDF/GO/*q*-PMMA-*b*-PDMAEMA blend solution was prepared by mixing a PVDF (MW: 534,000) solution (16 wt%) in *N*,*N*-dimethylformamide and acetone (v:v = 6:4) with 1.5 wt% of GO and 0.5 wt% of *q*-PMMA-*b*-PDMAEMA (EBC, BBC, or OBC) at 50 °C. Each as-prepared solution was placed in a syringe with a metal needle (inner diameter of 0.5 mm). The PVDF/GO/*q*-PMMA-*b*-PDMAEMA nanofibers were then deposited on aluminum foil fixed to a rotating cylinder (diameter = 9 cm, speed = 800 rpm). Polymorphism and morphology of nanofiber depend on electrospinning conditions including mainly flow rate, applied voltage, and distance from the needle to the collector^43^, thus, optimized electrospinning condition was determined by prior experiments. The optimum electrospinning conditions for the PVDF/GO/*q*-PMMA-*b*-PDMAEMA solutions were investigated in the range of applied voltage (10–30 kV) and flow rate (0.1–1.5 ml/h) with tip-to-collector distance at 15 cm. There is no difference among three kinds of *q*-PMMA-*b*-PDMAEMA (0.5 wt%), while GO (1.5 wt%) play an important role in electrospinning PVDF/GO/*q*-PMMA-*b*-PDMAEMA solutions. Therefore, optimum electrospinning condition was determined equally as 0.2 mL/h of flow rate and 25 kV of applied voltage at 15 cm of tip-to-collector distance as no beaded nanofibers appeared and electrospinning stability and uniformity of the nanofiber was high. Pristine PVDF/GO nanofibers were also prepared by the same procedure without any *q*-PMMA-*b*-PDMAEMA additives for comparison. The as-prepared nanofibers were dipped into 2 wt% PVA solution in deionized water for 1 min, then soaked in methanol for 24 h to crosslink PVA, and then dried at 60 °C to give PVDF/GO/q-PMMA-b-PDMAEMA@PVA nanofibers.

### Characterization of PVDF/GO/q-PMMA-b-PDMAEMA@PVA nanofibers

The characteristics of the PVDF/GO/*q*-PMMA-*b*-PDMAEMA@PVA nanofibers were evaluated using analytical techniques. Field emission scanning electron microscopy (FESEM, SUPRA 55VP, Carl Zeiss, Oberkochen, Germany) and transmission electron microscopy (TEM, Titan 80–300, FEI, Hillsboro, OR, USA) were examined for the morphology of the nanofibers. The average nanofiber diameter was determined by measuring nanofibers (n = 30) using ImageJ 1.43 u software (National Institutes of Health, Bethesda, MD, USA) in each FESEM image. The thickness of each nanofiber was measured using a dial thickness gauge (Mitutoyo, Japan). To investigate the surface hydrophilicity, time-dependent water contact angles were measured by the sessile drop method using a contact angle analyzer (Phoenix 300, Surface Electro Optics Co. Ltd, Seoul, Korea) for 10 s. The amounts of PVA coated on the nanofibers were calculated by measuring weights before and after PVA coating. Fourier transform infrared (FT-IR) spectra of the nanofibers were recorded on a Nicolet iS10 spectrometer (Thermo Scientific, Waltham, MA, USA). The fractions of β-phase (F(β)) in the nanofibers were calculated from FT-IR spectra following the Lambert-Beer law, which is the equation (1)^44^:

1 $${\rm{F}}({\rm{\beta }})={A}_{\beta }/(({K}_{\beta }/{K}_{\alpha }){A}_{\alpha }+{A}_{\beta })$$

where *A*_*α*_ and *A*_*β*_ are the adsorption at 766 and 840 cm^−1^, and *K*_*β*_ (7.7 × 10^4^ cm^2^/mol) and *K*_*α*_ (6.1 × 10^4^ cm^2^/mol) are the adsorption coefficients at the respective wavenumber.

### Evaluation performance of water permeation

The flow pore diameters of the nanofibers were evaluated by a capillary flow porometer (CFP-1500AE, Porous Materials Inc., Ithaca, NY, USA). The porosity (*ε*) was calculated by a gravimetric method using the equation (2):

2 $$\varepsilon \,( \% )=({m}_{w}-{m}_{d})\times 100/({\rho }_{w}Al)$$

where *m*_*w*_ is the weight of the wet nanofibers in water, *m*_*d*_ is the weight of the dry nanofiber, *ρ*_w_ is the density of water, *A* is the effective area of the nanofiber, and *l* is the nanofiber thickness. The permeation fluxes of the nanofibers were determined using a dead-end filtration system (transmembrane pressure (TMP) = 0.93 bar) at room temperature. Deionized water (1.0 L) was used in the permeation flux tests and the permeation flux (*J*_*w1*_) was estimated based on the equation (3):

3 $${J}_{w1}=V/(S\times t)$$

where *V* is the total permeation volume, *S* is the total permeation area (m^2^), and *t* is the total permeation time (min).

### Evaluation of anti-fouling activity: Fouling reversibility

Bovine serum albumin (BSA, Sigma Aldrich, St. Louis, MO, USA) was chosen as a model protein to cause fouling of the nanofibers. BSA filtration tests were conducted using the following procedure (TMP = 0.93 bar): 1) Deionized water was filtered through each nanofibers (PVDF/GO@PVA, PVDF/GO/EBC@PVA, PVDF/GO/BBC@PVA and PVDF/GO/OBC@PVA) until the flux remained stable prior to BSA solution injection. 2) The BSA solution (1.5 L, 1 g/L in deionized water at pH 6.3) was applied to each nanofibers and the flux of the BSA solution (*J*_*p*_) was measured. 3) Each nanofibers was backwashed with 1 L of deionized water and rinsed for 20 min at 170 rpm using a shaker (JSSI-100T, JS Research Inc., Gongju, Korea). 4) Pure water was reinjected, and then the pure water flux (*J*_*w2*_) was measured to determine the fouling reversibility. The flux recovery ratio (FRR) was calculated using equation (4):

4 $$\text{FRR}\,( \% )=({J}_{w2}/{J}_{w1})\times 100\,$$

The total (R_t_), reversible (R_r_), and irreversible (R_irr_) fouling ratios were calculated using equations (5–7):

5 $${{\rm{R}}}_{t}( \% )=(1-{J}_{p}/{J}_{w1})\times 100\,$$

6 $${{\rm{R}}}_{r}( \% )=({J}_{w2}-{J}_{p})/{J}_{w1}\times 100\,$$

7 $${{\rm{R}}}_{irr}( \% )=({J}_{w1}-{J}_{w2})/{J}_{w1}\times 100\,$$

### Evaluation of anti-fouling activity: Antibacterial activity

Antibacterial tests were performed with PVDF/GO@PVA, PVDF/GO/EBC@PVA, PVDF/GO/BBC@PVA and PVDF/GO/OBC@PVA nanofibers using two types of bacteria, Gram-negative *Escherichia coli* (ATCC 11105) and Gram-positive *Staphylococcus aureus* (ATCC 6538), and three methods (agar diffusion, dynamic contact, and LIVE/DEAD bacterial cell viability assay). First, the circular nanofibers (diameter = 1 cm) were put on TSA agar plates containing each bacterial inoculum. Then, the plates were incubated at 37 °C for 18 h and the zone of inhibition was observed. For dynamic contact tests, the nanofibers (diameter = 2 cm, weight = 8.39 ± 0.16 mg) were added to 30 mL of each bacterial solution (initial *E*. *coli* concentration = 4.97 × 10^5^ CFU/mL, initial *S*. *aureus* concentration = 8.48 × 10^5^ CFU/mL). The mixtures were shaken at 150 rpm and 25 °C using a shaker (JSSI-100T, JS Research Inc., Gongju, Korea) for the desired contact time (60, 120 and 240 min). A plate counting method was used for enumeration of bacteria in suspensions after incubation at 37 °C for 24 h. The removal capacity of bacteria per unit mass (CFU/mg) of the nanofibers was calculated as follows:

8 $$Bacteria\,removal\,capacity=[({C}_{0}-{C}_{s}+{C}_{a})/{C}_{0}]/M$$

where *C*_0_ is the initial bacteria concentration in solution (CFU/mL), *C*_*s*_ is the suspended bacteria concentration in the solution after reaction (CFU/mL), *C*_*a*_ is the detached bacteria concentration from the nanofibers after vigorously glass bead vortexing for 1 min in an autoclaved phosphate buffer solution (PBS) after the contact time (CFU/mL), and *M* is the mass of the nanofibers (mg). Finally, live and dead bacterial cells on the nanofibers were stained by LIVE/DEAD BacLight bacterial viability kit (L13152, Molecular Probes, Leiden, Netherland) and clearly observed by confocal laser scanning microscopy (CLSM, LSM 700, Carl Zeiss, Jena, Germany) after bacterial solution injection (50 µL) on each nanofibers (1 cm^2^) for 4 h at 37 °C.

## Electronic supplementary material

Supplementary information
