# Supplementary material for: Quaternized Amphiphilic Block Copolymers/Graphene Oxide and a Poly(vinyl alcohol) Coating Layer on Graphene Oxide/Poly(vinylidene fluoride) Electrospun Nanofibers for Superhydrophilic and Antibacterial Properties
Source: Sci Rep. 2019 Jan 23;9:383. doi: 10.1038/s41598-018-36479-w (PMC6344507; doi:10.1038/s41598-018-36479-w)
Supplement: Supplementary file 1 — Supplementary information [file 41598_2018_36479_MOESM1_ESM.docx]

Quaternized Amphiphilic Block Copolymers/Graphene Oxide and a Poly(vinyl alcohol) Coating Layer on Graphene Oxide/Poly(vinylidene fluoride) Electrospun Nanofibers for Superhydrophilic and Antibacterial properties

Jeong-Ann Park^1,2^, Kie Yong Cho^3*^, Chee Hun Han^4,5^, Aram Nam^4,5^, Jae-Hyun Kim^4^,

Sang-Hyup Lee^4,5^, Jae-Woo Choi^4,6*^

*^1^Environmental Functional Materials and Water Treatment Laboratory, Department of Rural Systems Engineering, Seoul National University, Seoul 08826, Republic of Korea*

*^2^ Research Institute of Agriculture and Life Sciences, Seoul National University, Seoul 08826, Republic of Korea*

*^3^ Artie McFerrin Department of Chemical Engineering, Texas A&M University, College Station, TX 77843-3122, United States.*

*^4^ Center for Water Resource Cycle Research, Korea Institute of Science and Technology, Hwarangno 14-gil 5, Seongbuk-gu, Seoul 02792, Republic of Korea*

*^5^ KU-KIST Green School, Graduate School of Energy and Environment, Korea University, 145 Anam-ro, Seongbuk-gu, Seoul 02841, Republic of Korea*

*^6^ Division of Energy & Environment Technology, KIST School, Korea University of Science and Technology, Seoul 02792, Republic of Korea*

Fig. S1. GPC curves of the PMMA macroinitiator and the PMMA-*b*-PDMAEMA diblock copolymer.

**Table S1**

Results of agar diffusion method using the nanofibers.

| Nanofibers | Bacteria | Growth assessment |  |
| --- | --- | --- | --- |
|  |  | Zone of inhibition (mm) | Growth under nanofibers |
| PVDF/GO@PVA | *E. coli* | 0.0 | Well-growth |
|  | *S. aureus* | 0.0 | Well-growth |
| PVDF*/*GO/EBC@PVA | *E. coli* | 0.0 | Mid-growth |
|  | *S. aureus* | 0.0 | Well-growth |
| PVDF*/*GO/BBC@PVA | *E. coli* | 0.0 | No growth |
|  | *S. aureus* | 0.0 | Mid-growth |
| PVDF/GO/OBC@PVA | *E. coli* | 0.0 | No growth |
|  | *S. aureus* | 0.0 | No growth |
